# Somatosensory Integration and Masking of Complex Tactile Information: Peripheral and Cortical Contributions

**DOI:** 10.3390/brainsci10120954

**Published:** 2020-12-09

**Authors:** Steven R. Passmore, Niyousha Mortaza, Cheryl M. Glazebrook, Bernadette Murphy, Timothy D. Lee

**Affiliations:** 1 Department of Kinesiology, McMaster University, Hamilton, ON L8S 4K1, Canada; scapps@mcmaster.ca; 2 Research Department, New York Chiropractic College, Seneca Falls, NY 13148, USA; 3 Faculty of Kinesiology and Recreation Management, University of Manitoba, Winnipeg, MB R3T 2N2, Canada; mortazan@myumanitoba.ca (N.M.); Cheryl.Glazebrook@umanitoba.ca (C.M.G.); 4 Faculty of Health Sciences, Ontario Tech University, Oshawa, ON L1G 0C5, Canada; Bernadette.Murphy@ontariotechu.ca

**Keywords:** masking, response competition, Morse code, paresthesia, tactile learning, vibrotactile stimulus, somatosensory evoked potentials

## Abstract

Nerve paresthesia is a sensory impairment experienced in clinical conditions such as diabetes. Paresthesia may “mask” or “compete” with meaningful tactile information in the patient’s sensory environment. The two objectives of the present study were: (1) to determine if radiating paresthesia produces a peripheral mask, a central mask, or a combination; (2) to determine if a response competition experimental design reveals changes in somatosensory integration similar to a masking design. Experiment 1 assessed the degree of masking caused by induced radiating ulnar nerve paresthesia (a concurrent non-target stimulus) on a vibrotactile Morse code letter acquisition task using both behavioral and neurophysiological measures. Experiment 2 used a response competition design by moving the radiating paresthesia to the median nerve. This move shifted the concurrent non-target stimulus to a location spatially removed from the target stimuli. The task, behavioral and neurophysiological measures remained consistent. The induced paresthesia impacted letter acquisition differentially depending on the relative location of meaningful and non-meaningful stimulation. Paresthesia acted as a peripheral mask when presented to overlapping anatomical stimulation areas, and a central mask when presented at separate anatomical areas. These findings are discussed as they relate to masking, subcortical, and centripetal gating.

## 1. Introduction

In the past decade, the focus of most haptic, tactile, or touch research has been to maximize the human operator interface to improve safety and efficiency in the workplace for fully, sensory-enabled workers [1]. For example, highway rumble strips create full-body vibration to alert drivers that their current location is deviating from the center of the roadway. Rumble strip placement is selected strategically in areas of risk where driver alertness is crucial [2]. Tactile, or haptic, feedback and vibrotactile stimulation have been extensively used in recent technology to alert and communicate with users. For example, tactile stimulation is considered a less disruptive (compared to other modalities) means to identify incoming signals from personal communication devices. A primary goal of tactile information processing is to identify the signal from the noise, that is, to identify the target stimulation relevant to the task. Tactile cues compete for attentional resources in the presence of multiple stimuli and modalities.

In order to study tactile attention, researchers have used the technique of sensory masking by creating a tactile mask. A tactile mask is created by the presentation of an additional tactile stimulus that interferes with the detection of the target tactile stimulus [3]. The technique of dual stimulation of the touch modality is useful to ascertain the source of the masking. This technique explores the relative contributions of interference from competing input at the level of the sensory receptors (peripheral mask) versus at the level of overload in cortical processing (central mask). Competing stimuli may be two meaningful stimuli (e.g., tactile Morse code letters) or a combination of meaningful and not meaningful (e.g., noxious) stimuli.

For example, in 1994, Apkarian, Stea, and Bolonowski found tactile sensitivity to be suppressed by heat delivered to the point of creating pain [4]. In their study, both tactile- and pain-provoking stimuli were presented to the base of the thumb of the right hand. Participants detected the presence of vibrotactile stimuli with and without concurrent heat-induced pain. The authors interpreted tactile-sensitivity suppression with concurrent pain to be evidence of a “gating” mechanism in the tactile system. Based on the afferent tactile pathway entering the dorsal column tracts of the spinal cord and synapsing in the dorsal column nuclei in the brainstem, they believed the disruption of tactile perception likely occurred centrally. However, Apkarian et al. [4] only considered the detection of simple vibrotactile stimuli and not the perception of complex stimuli. Spence and Gallace (2007) [5] acknowledged the importance of looking at intramodal attention, with the suggestion of exploring ecologically-valid testing conditions to understand better the impact of concurrent distracting stimuli that a participant may need to ignore to perform a task successfully.

The study of complex stimuli is important to understand better the communication of sophisticated information typically processed cortically. Humans will use a combination of strategies to complete complex vibrotactile-driven tasks. For example, they will orient their attention endogenously to the sense of touch and then await exogenously-driven tactile stimuli. The need for exploration of higher-level cognitive attention studies of complex tactile information has been identified as a priority for future research [5]. The primary application of complex tactile information delivery and reception is for use when other sensory modalities are occupied or impaired. For example, workers on a noisy factory floor may need information about their task performance, or soldiers in a military context may require information about their position or details regarding their enemy when other forms of communication are not safely possible.

Craig (1983) [6] determined that perturbation of a finger other than the finger required for a tactile discrimination task does not sufficiently distract the participant from the task’s cognitive demands. While changing the target finger used was unsuccessful in impacting tactile discrimination ability, when stimulation was moved to an alternate location on the same finger, then performance was impacted. Kekoni et al. (1990) [7] found a decreased masking effect when the mask’s spatial location was switched to the base of the finger, and the to-be attended stimuli was shifted to the fingertip. They determined that a mask is more salient when placed over an area of dense innervation (fingertip). Their methods were unable to discern specifically whether the masking was strictly due to increased density of innervation or possibly the larger cortical representation of the stimulated brain area. A limitation of Craig’s [6] results is the potential role of the same or separate peripheral innervation of the regions stimulated by the mask and the to-be-attended stimuli. If Craig’s [6] data were reinterpreted with the concept of innervation location in mind, it would appear in general that a mask was less salient when anatomical locations with different peripheral innervations were presented with masking and meaningful stimuli, respectively. Kekoni et al. [7] found that the masking effect was decreased when either longitudinal distance (from the wrist to the fingers) or transverse distance (from the base of the second digit to the base of the fifth digit) between the mask stimulus and target stimulus on the palm of the hand was increased. Based on their findings, they hypothesized that masking might have a central, as opposed to a peripheral mechanism.

Our previous work successfully applied a perturbation of transcutaneously-induced paresthesia as a non-meaningful secondary stimulus that impacts the tactile system [8]. Participants in the acquisition phase of learning complex vibrotactile information (Morse code letters) demonstrated longer total response times and poorer accuracy scores when paresthesia was presented concurrently to the same peripheral nerve innervation region as the target letter stimuli was presented to. These results were explained from the viewpoint of masking, in that the paresthesia stimulus acted to mask the meaningful complex vibrotactile afferent input. In contrast, no detriment to the acquisition of the Morse code letters was noted when a response competition design, where the paresthesia perturbation was applied to a region anatomically separate from the complex target stimuli, was used. The conclusions from this research are consistent with the findings of Craig’s study (1995) [9]. That is, neurophysiological studies are needed to further the understanding of the way in which complex tactile information is reflected in the nervous system and at what level it is processed.

Somatosensory evoked potentials (SEPs) reflect the neuroplasticity associated with a perceptual [10] or motor task [11,12,13] when baseline SEPs are compared to SEPs recorded following the practice of a perceptual, sensory, or motor task [14]. A pre- and post-test experimental design can be used to avoid inadvertently masking the tactile system while utilizing the SEPs technique. Pellicciari et al. (2009) [10] compared SEPs recordings in an older and young sample, pre-and post-exposure to paired-associative stimuli. While neuroplasticity may take place in both populations with learning, the patterns and underlying structures reflecting neuroplastic changes appeared to differ. Murphy et al. (2003) [11] used pre-and post-task SEPs as a neurophysiologic measure for the plasticity-related to motor output. Ten individuals had SEPs recorded at baseline, then immediately after a 20 min repetitive-typing task. Attenuation of the N13 peak, N14-18 complex, and N30-P40 complex all occurred immediately following a typing task. Thus, SEPs is an ideal tool to give insight into changes in cortical processing associated with task practice and is used in the current experiments to study the changes in cortical processing as a result of learning a Morse Code task with and without induced paresthesia. 

In the current experiments, similar to our previous study [8], to validate and quantify the applied sensory perturbation (i.e., induced paresthesia), two-point discrimination and monofilament pressure sensitivity tests were used as sensory measurements. In the monofilament test, monofilaments of different thicknesses were used to apply pressure on the skin to determine the perception of sensation and to assess general pressure detection capacity. The two-point discrimination test was used to look at the resolution of the tactile sense. That is, the higher tactile resolution is present if the two-points are closer together and still perceived as two separate contact points, and lower if the two separate contact points needed to be farther apart to be perceived as two separate points and not a single point.

The purpose of the present two experiments was to determine if the acquisition of complex tactile-driven information elicits changes in somatosensory integration, and if those changes can be perturbed by concurrent, non-meaningful stimulation using a pre-post SEPs stimulation design. Experiment one investigated the masking effects of a local perturbation, concurrent with complex tactile stimulation. Experiment two investigated the response competition between a concurrent tactile perturbation to a region of separate peripheral innervation from the complex tactile target.

## 2. Experiment 1

The goal of experiment one was to resolve whether the masking of complex tactile information reflects competing input at the level of the sensory receptors (peripheral mask) or at the cortical level as a processing overload (central mask), or a combination of both. To test masking in a within-participant study, somatosensory evoked potentials were recorded at baseline and following the acquisition of complex tactile information (vibrotactile Morse code letters). Letter acquisition was performed separately under conditions of induced paresthesia as a masking perturbation and under no perturbation. We expected that the results of the behavioral performance measures of the current experiment would replicate previous findings [8], that is, response times should increase, and accuracy should decrease with perturbation. Hence, if differences in the SEPs component amplitudes were found between paresthesia (non-meaningful stimulation) and no paresthesia (no sensory stimulation condition), then both peripheral and central masking had occurred. This finding indicates that both peripheral and cortical (related to N20, N24) factors were involved in the observed complex information masking. Increases or attenuation of specific SEPs peaks will reveal information about where the perturbation impacted information processing. The processing locations were predicted based on peripheral limitations or the suspected cortical generators of the impacted SEPs peak locations.

### 2.1. Method

#### 2.1.1. Participants

Twelve participants (7 women, age range 19–31 years, M = 22.3 years, SD = 3.7 years) volunteered to take part in the experiment. All were naïve regarding the purpose of the experiment, and 11 were right-handed by self-report. All had normal, or corrected-to-normal vision, and none reported any history of gross neurological sensory deficits. Approval by the Ontario Tech University (07-072) and McMaster University Ethics Boards was granted, and informed consent was obtained from all participants. This study was carried out according to the ethical standards set out by the Declaration of Helsinki for the use of humans in experimental studies.

All participants were sensory tested for tactile sensitivity [15], then asked to rate their knowledge of Morse code on a 5-point Likert scale [16], where 5 represented no knowledge, and 1 represented Morse code fluency. Only participants with no knowledge of Morse code (a score of 5) proceeded.

#### 2.1.2. Design

The study was a within-participant design that was divided into two phases and conducted over two days. Each day included six acquisition blocks of trials to quantify changes in performance over the six practice blocks. Day one consisted of the acquisition of one letterset, while day two consisted of the acquisition of another letterset containing all new letters. Lettersets were counterbalanced between participants to avoid order effects and any possible systematic effects of letter difficulty. Participants were also assigned alternately to either receive peripheral nerve stimulation on day 1 or no stimulation first. The experimental design was counterbalanced so that all participants would eventually proceed to the condition opposite that which they were assigned initially.

#### 2.1.3. Apparatus and Materials

Participants wore blindfolds and sat in a comfortable chair approximately 150 cm away from a computer monitor. Participants supinated their right hand for both two-point discrimination (Touch-Test two-point discriminator, Northcoast Medical Inc., Morgan Hill, CA, USA) and monofilament pressure sensitivity (Touch-Test sensory evaluators, Northcoast Medical Inc, Morgan Hill, CA, USA). Specifically, sensory testing occurred over the palmar surface of the distal aspect of the fifth digit of their right hand. Sensory testing was repeated prior to the component of the experiment that included induced paresthesia.

#### 2.1.4. Non-Task-Specific Perturbation (Induced Paresthesia)

Half of the participants received stimulation from a DS7A Constant Current Stimulator (Digitimer Ltd., Welwyn Garden City, Hertfordshire, UK) for the duration of the acquisition phase of the experiment on day 1. Stimulation was delivered transcutaneously through 7 mm Ag/AgCl disposable, adhesive electrodes (Hydrospot from Physiometrix, Inc., Billerica, MA, USA). Two electrodes were placed on the surface of the dorsal medial aspect of the arm, 2 cm proximal to the ulnar notch, over the predicted course of the ulnar nerve. Custom LabVIEW (National Instruments, Austin, TX, USA) software externally triggered the constant current stimulator, with a pulse duration of 200 μs, an interstimulus interval of 10 milliseconds (ms), and a voltage edge of 0.2 V.

Similar to the method used in our previous study [8], the measurement of discrimination thresholds (sensory, radiating, premotor; see [17] for more details) was performed to establish settings for the constant current stimulator premotor threshold. The premotor threshold was the most intense stimulation delivered before motor contraction occurred, and was the intensity used for the duration of the experiment. For comparison to baseline, participants had their sensory testing, including monofilament pressure test and two-point discrimination test, repeated on the day they received paresthesia stimulation from the constant current stimulator.

#### 2.1.5. Vibrotactile Task

Participants started with their right hand in a prone position with their elbow bent to 90° and supported by an armrest, while the distal aspect of their left index finger rested atop the “V” key (used as the home position) on a QWERTY keyboard placed on their lap. The distal aspect of their right hand’s 5th digit rested atop a 12 millimeters (mm) diameter × 3.4 mm thick, 200 Hz vibrating disc motor (Solarbotics Ltd., Calgary, AB, Canada). Custom E-Prime software (Psychology Software Tools, Inc., Pittsburgh, PA, USA) guided participants through the experiment, recorded dependent variables, and triggered the vibrotactile motor to create patterns representing Morse code.

Presentation of Morse code letters was achieved through patterns of vibrations (long and short pulses with spaces in between) using only letters with no more than four components, and no less than 3 components (e.g., Z = “- - . .”; R = “. - .”). Pulse durations of 750 ms represented “dash” (-) signals, while durations of 250 ms represented “dot” (.) signals. Spaces between letters were set to durations of 125 ms [8,18].

Participants were exposed to a random presentation of 8 different Morse code letters until each letter was presented six times (48 total letter presentations). The computer screen provided a subset of 8 possible letter options and participants were then asked to indicate which letter had been presented by pressing the appropriate key on an alphanumeric keyboard with their left index finger. Augmented feedback regarding response accuracy was provided—the correct letter was presented visually in its roman alphabetic form on the screen. The augmented information was provided retroactively to facilitate learning compared to providing the correct information before a response was made [19]. The participant self-selected when to depress the spacebar following feedback presentation and returned their left index finger to the home “V” key in order to move on to the next trial (stimulus presentation). Total response time (TRT) was the duration between when the screen requesting a letter selection appeared (immediately following vibrotactile presentation) until the response key was selected and depressed. Selection accuracy was the number of correct (1) or incorrect (0) responses.

#### 2.1.6. SEPs Stimulation and Recording

Participants were seated in a comfortable chair, in a quiet room with their eyes open and the lights off. Stimulation electrodes were placed over the suspected course of the ulnar nerve of the right arm. Stimuli delivery occurred at 103% of the motor threshold, applied using a constant current stimulator (Digitimer Ltd., Hertfordshire, UK) at a rate of 4.98 Hz, a rate selected to maximize the accuracy of the N24 peak measurement [20,21].

SEPs were collected in trial blocks of 1500 sweeps. EEG signals were band-passed filtered (3–1000 Hz) and amplified (gain 100,000). Custom LabVIEW (National Instruments, Austin, TX, USA) software controlled the data acquisition, signal averaging, and graphing functions for data analyses. The EEG signals were digitized at a sample rate of 5000 samples per second and were recorded with a sweep length of 55 ms (5 ms pre-stimulus and 50 ms post-stimulus). All surface recording electrodes were 10 mm disc, 2 mm hole, gold cup EEG electrodes (Grass Technologies, West Warwick, RI, USA) and were placed according to the International Federation of Clinical Neurophysiologists (IFCN) recommendations [22]. IFCN recommended recording sites include electrode placement on ipsilateral Erb’s point, and 2 cm posterior to contralateral central and frontal scalp sites C3/C4 (Cc’) and F3/F4 (Fc’), respectively. Additionally, one anterior cephalic electrode was placed 6 cm anterior to Cz and 2 cm contralateral to the side of stimulation, as identified by Rossi et al. (2003) [23]. All electrodes were referenced to the ipsilateral earlobe, while a ground electrode was placed in the participant’s mouth, tucked behind the lower lip.

Peripheral (N9), brainstem (N18), and cortical (N20, N24, and N30) SEPs to ulnar nerve stimulation were recorded before and after letter acquisition. Measurement and identification of SEPs peak amplitudes was conducted according to the IFCN guidelines [24]. All peaks were analyzed with the exception of peak N9, which was simply used as analysis exclusion criteria. The peripheral N9 peak was used to assess the stability of the signal of the peripheral afferent volley at the level of the brachial plexus. Participants with N9 peaks with more than 10% variability were to be excluded from the final SEPs waveform analysis. No participants in the current study met this criterion.

The SEPs stimulation and concurrent data collection occurred at baseline and following vibrotactile letter acquisition. Neurophysiological changes and behavioral data can be examined in tandem to create an understanding of whether learning is evident from performance improvement and changes in central nervous system electrical activity [14]. The suspected generator of a SEP component may be used to provide insight into where central changes occurred.

#### 2.1.7. Data Analysis

Normal distribution of results of the primary dependent variables (i.e., SEPs outcome measures) were assessed and confirmed using Kolmogorov–Smirnov tests.

Sensory data analysis: Paired Student’s t-tests were performed for both two-point discrimination, and monofilament testing to compare sensory data collected at baseline to sensory data collected during paresthesia stimulation.

Behavioral data analysis: Acquisition data consisting of mean total response time, and mean accuracy were each compared using within group analysis of variance (ANOVA) models. The designs were separate 2 (sensation: paresthesia, no paresthesia) × 6 (trial block: acquisition 1–6) ANOVA models with repeated measures on both factors. Significant main effects and interactions were reported, *p* < 0.05.

SEPs data analysis: SEPs data, consisting of the mean amplitude of analyzed peaks, was compared between the initial baseline, and following the 6 acquisition blocks. The design was a separate 2 (sensation: paresthesia, no paresthesia) × 2 (trial time: baseline, post-learning) ANOVA model, with repeated measures on both factors. Significant main effects and interactions were reported for each pre-identified peak. Significance for all statistical measures was set at *p* < 0.05. Tukey’s post-hoc analysis was applied for the interpretation of results with more than two means.

### 2.2. Results

#### 2.2.1. Sensory Testing

Significant differences were found for both two-point discrimination, *t* (11) = −3.08; *p* = 0.01, and monofilament pressure testing, *t* (11) = −8.48; *p* < 0.001. Paresthesia stimulation led to impaired two-point discrimination (M = 3.58) compared to no stimulation (M = 2.75). Paresthesia stimulation also led to a decrement in monofilament testing (M = 3.67), compared to no stimulation (M = 2.90).

#### 2.2.2. Behavioral Performance

Participants were presented with eight response alternatives when selecting which letter they believed was presented on that trial. Thus, there was a 12.5% chance of guessing the correct response on each trial.

**Figure 1:**
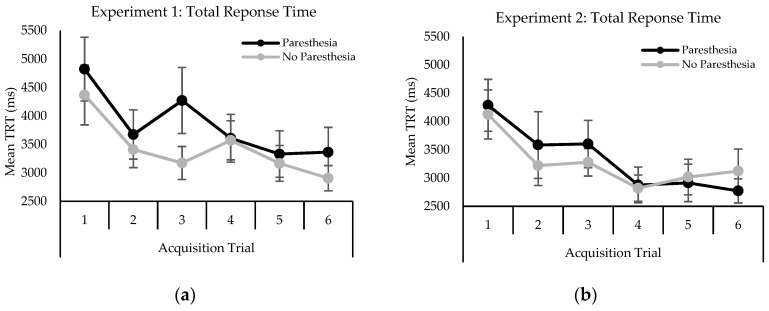
Mean accuracy (percent correct) by condition during acquisition experiments 1 (**a**) and 2 (**b**).

Analysis of mean accuracy yielded a main effect for trial, F (5,55) = 9.62; *p* <0.001, η^2^ = 0.329. Accuracy increased as the trial number increased (Figure 1a). Post-hoc analysis revealed that accuracy on trial 1 (M = 15.63) was significantly less than all other trials, and trial 2 (M = 31.25) was significantly less than trial 6 (M = 45.83). There was also a main effect for sensory condition, F (1,11) = 5.32; *p* < 0.001, η^2^ = 0.156. Letter identification accuracy decreased in the presence of paresthesia (M = 29.86%) compared to no paresthesia (M = 40.28%).

**Figure 2:**
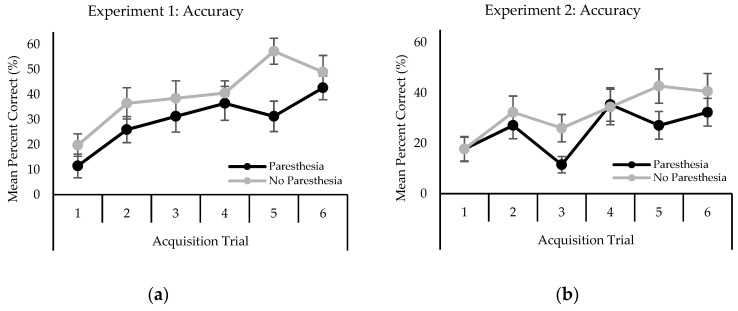
Mean total response times (TRT in Milliseconds) by condition during acquisition in experiment 1 (**a**) and 2 (**b**). Error bars represent standard error.

Analysis of mean TRT revealed a main effect for trial, F (5,55) = 6.98; *p* < 0.001, η^2^ = 0.132. TRT decreased as the trial number increased (Figure 2a). Post-hoc analysis revealed that TRT on trial 1 (M = 4595 ms) was significantly longer than all other trials.

#### 2.2.3. SEP Amplitude

**Figure 3:**
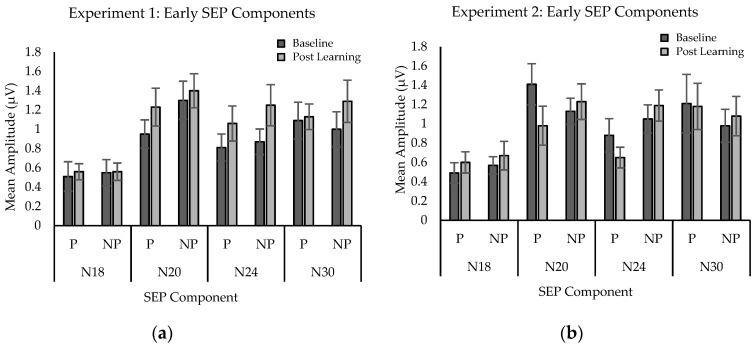
Mean early SEPs component amplitude (in microvolts) by condition in Experiments 1 (**a**) and 2 (**b**) at baseline and post-learning. P, with paresthesia; NP, without paresthesia. Error bars represent standard error.

**Figure 4:**
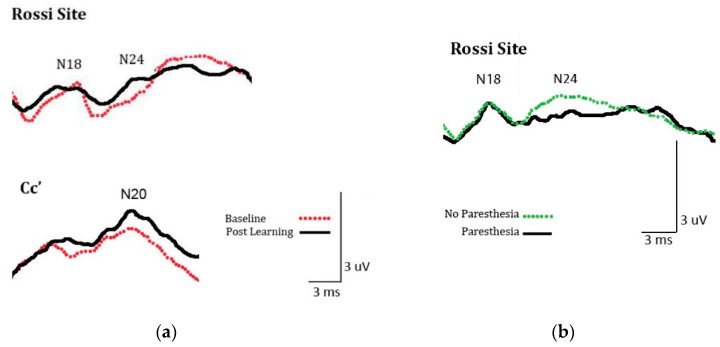
(**a**) An example of the significant somatosensory evoked potentials (SEPs) amplitude increase (N20, N24) from the averaged waveform of a single participant from experiment 1 between baseline and post tactile learning; (**b**) An example of the significant SEP amplitude attenuation (N24) from the averaged waveform of a single participant from experiment 2 between paresthesia, and no paresthesia conditions.

Significant differences were found for SEPs peaks N20 and N24, but no significant differences were found for the other peaks measured (Figure 3a). Peak N20 analysis included 11 participants as one participant was excluded due to a loose electrode. The analysis yielded a main effect for time, F (1,10) = 14.49; *p* = 0.003, η^2^ = 0.052. Mean amplitude in microvolts was significantly greater at post-learning (M = 1.315) than at baseline (M = 1.122). Peak N24 analysis also revealed a main effect for time, F (1,11) = 6.58; *p* = 0.026, η^2^ = 0.142. Mean amplitude was significantly greater post-acquisition (M = 1.153) than at baseline (M = 0.843). Figure 4a illustrates the SEPs average waveform amplitude increases (N20, N24) from baseline to post-acquisition for a single participant.

### 2.3. Discussion

The behavioral results replicate previous work [8] where, compared to when no paresthesia is present, total response time increased, and accuracy decreased when paresthesia was applied to the ulnar nerve, which was also the innervation for the site of vibrotactile Morse code letter presentation.

The N20 SEP amplitude increased in response to the acquisition of complex vibrotactile information. The N20 SEP peak is recorded over the parietal cortex and is generated in the primary somatosensory cortex (S1), representing neurons within Brodmann’s area 3b [22,25,26]. The N20 is known to respond to tactile stimulation [27]. The N24 peak also increased in amplitude, a site considered to reflect activity in pathways involving the cerebellum and S1 [28]. The SEPs peak changes were similar for both conditions, with no differences between sensory conditions (with paresthesia versus without paresthesia).

The converging agreement between behavioral and neurophysiological measures provides evidence for complex tactile learning. Behavioral performance improved across the six acquisition blocks, demonstrated by reduced time to respond and improved accuracy. The SEPs component amplitude increased after acquisition compared to baseline for both conditions, indicating tactile learning in general. The differences between sensory conditions in the behavioral results, but not in SEPs component amplitude, indicate that the detriment in performance can be explained by a peripheral masking effect.

## 3. Experiment 2

Experiment two aimed to resolve whether a response competition design reveals changes in somatosensory integration similar to the peripheral masking design from experiment one. Response competition is thought to occur centrally when resolving competing stimuli, while masking has a peripheral mechanism. When compared to baseline, changes in the amplitude of SEPs waveforms in a response competition design could reveal the presence of any central processing conflicts, and possibly where they occur within the nervous system. To achieve this goal, somatosensory evoked potentials were recorded at baseline and following the acquisition of complex tactile information (vibrotactile Morse code letters) using a within participant design. Letter acquisition was performed separately under conditions of induced paresthesia and under no perturbation. To create a response competition design, an anatomical region with innervation distinct from the location of the target stimuli presentation was selected. The participant was instructed to actively ignore the secondary stimulus and to focus on the detection and interpretation of the complex target stimuli (Morse code letter). If only the SEPs analyses demonstrate between-sensory condition differences, then the results reflect a central resolution of a response competition paradigm. Conversely, if there are no differences in the SEPs analyses, then a subcortical mechanism is responsible for tactile response competition.

### 3.1. Method

#### 3.1.1. Participants

Twelve participants (4 women, age range 20–28 years, M = 22.3 years, SD = 2.3 years) volunteered for experiment two. All were naïve regarding the purpose of the experiment, had normal or corrected-to-normal vision, and none reported any history of gross neurological sensory deficits. Approval by the University of Ontario Institute of Technology (UOIT) (07-072) and McMaster University ethics boards were granted, and informed consent was obtained from all participants. This study was carried out according to the ethical standards set out by the Declaration of Helsinki for the use of humans in experimental studies.

#### 3.1.2. Apparatus, Materials, Design, Procedure, and Analysis

All procedures and equipment were the same as experiment 1, with the exception of the change in location of the transcutaneous electrode placement used to create radiating paresthesia. These electrodes were moved to the anterior aspect of the forearm along the midline, over the predicted course of the median nerve; that is, 5 cm proximal to the carpal line. The DS7A Constant Current Stimulator (Digitimer Limited, Hertfordshire, UK) was set to deliver stimulation as in experiment one, with the exception that radiating paresthesia was now directed to the median nerve. The median nerve innervates the first, second, and third digits, and the lateral aspect of the fourth digit on the hand. The vibration motors used to create the complex vibrotactile stimuli (i.e., Morse code letters) were still placed under the palmer surface of the fifth digit. All statistical models for sensory testing, behavioral performance, and SEPs peak analyses were identical to experiment 1.

### 3.2. Results

#### 3.2.1. Sensory Performance

Paired Student’s *t*-tests yielded no significant differences. Median nerve paresthesia stimulation did not yield differences when compared to baseline for either two-point discrimination, or monofilament testing. No differences were found for both two-point discrimination, *t* (11) = 1.88; *p* = 0.09, and monofilament pressure testing, *t* (11) = 1.48; *p* < 0.09. Paresthesia stimulation led to equivalent two-point discrimination (M = 4.25) compared to no stimulation (M = 3.58). Paresthesia stimulation also yielded no difference in monofilament testing (M = 3.16) compared to no stimulation (M = 3.03).

#### 3.2.2. Behavioral Performance

As in experiment one, participants selected one of eight response alternatives for consideration based on which one of the eight possible letters they believed was presented. Therefore, there was a 12.5% chance of guessing the correct response on each trial.

Analysis of mean accuracy revealed a main effect for trial block, F (5,55) = 7.42; *p* < 0.001, η^2^ = 0.061. Accuracy increased as the trial block increased (Figure 1b). Specifically, post-hoc analysis revealed that accuracy on trial blocks 1 (M = 17.71) and 3 (M = 18.75) were significantly less accurate than trial blocks 4, 5 and 6 respectively (M = 34.90; M = 34.90; M = 36.46).

Mean TRT analysis also yielded a main effect for trial block, F (5,55) = 9.35; *p* < 0.001, η^2^ = 0.158 TRT decreased as trial block number increased (Figure 2b). Specifically, post-hoc analysis revealed that TRT on trial block 1 (M = 4206) was significantly slower than all other blocks of trials.

#### 3.2.3. SEPs Amplitude

A trend approaching significance was found for the N20 peak, while significant differences were found for SEPs peak N24. Figure 4b illustrates SEPs amplitude attenuation (N24) from the averaged waveform of a single participant between paresthesia, and no paresthesia conditions. No significant differences were seen for the other peaks measured (Figure 3b). Analysis of SEPs peak N20 yielded an interaction approaching significance F (1,11) = 3.90; *p* = 0.07, η^2^ = 0.079. The trend is consistent with a conflict between neural changes associated with learning (amplitude increases) and paresthesia (amplitude attenuation). When there was no paresthesia present, learning was demonstrated by a trend toward increased SEPs component amplitude (M = 1.13; to M = 1.24). The presence of paresthesia led a trend toward amplitude attenuation (M = 1.41; to M = 0.98). Analysis of SEPs peak N24 revealed a main effect for sensory condition F (1,11) = 7.37; *p* = 0.02, η^2^ = 0.401. The amplitude of the peak was attenuated in the presence of paresthesia stimulation (M = 0.764) compared to when no stimulation was present (M = 1.121).

### 3.3. Discussion

The behavioral results of experiment two are consistent with previous work [8]. That is, when paresthesia was applied to the median nerve, and the site of vibrotactile Morse code letter presentation was an ulnar innervated region, total response time and accuracy showed no differences compared to when no paresthesia was present.

In response to the acquisition of complex vibrotactile information, SEPs amplitude results demonstrated an interaction approaching significance for the N20 component. The analysis revealed a trend toward the waveform increasing in amplitude when no paresthesia was present, a finding that replicates experiment one above. However, when paresthesia was present, a trend toward waveform attenuation occurred after the acquisition of a complex letter task. One explanation for the lack of statistically significant difference for N20 between sensory conditions could be the small sample size (*n* = 12), in that the sample did not have enough power to show a statistically significant difference. In addition, significant attenuation of the N24 peak amplitude occurred. This site is considered to reflect the cerebellar role in somatosensory integration [28]. In addition, the attenuation occurred between sensory stimulation conditions, indicating that when the presence of paresthesia is in a separate region of innervation from the complex tactile stimuli that there is a different level of electrical activity in the structures responsible for the generation of the N24 peak.

The results from experiment 2 provided evidence for complex tactile learning. Compared to baseline, behavioral performance across the six acquisition trials improved in both sensory conditions. Attenuated precognitive cortical activity is reflected by the trend toward the differential impact of paresthesia stimulation of the N20 peak and the significantly attenuated N24 component amplitude. Thus, the SEPs findings indicate that a response competition paradigm effect occurred when dual stimulation was presented to separately innervated regions.

## 4. General Discussion

Both experiments demonstrated that participants could successfully acquire complex vibrotactile information. Tactile learning was challenging, as predicted by previous studies [29,30], with letter recognition accuracy less than 60%. This level of performance was consistent with previous tactile learning studies. Craig [30] noted a vibrotactile accuracy rate of 30–60% for letter learning. Similarly, Foulke and Broadbeck (1968) [29] taught Morse code letters using electrocutaneous stimulation and found a 59.4% accuracy rate. Learning was reflected in both sensory groups across blocks of trials in both behavioral variables (accuracy increase, TRT decrease), and in experiment one, by neurophysiological variables (the increase in cortical peak amplitude at N20 and N24). In experiment two, when the complex vibrotactile information was spatially separated from the perturbation of induced paresthesia, cortical measures recorded instead reflected competing afferent signals that attenuated cortical information processing.

With respect to the source of the peak amplitude changes in N20, in neurotypical participants, the N20 peak is the earliest marker of cortical processing in the primary somatosensory cortex following a somatosensory stimulus [23,31,32,33]. The primary somatosensory cortex lies in the posterior bank of the rolandic fissure representing Brodmann’s area 3b in the parietal lobe. The aforementioned anatomical region is the site of N20 peak generation [34] and is known to respond to contralateral tactile stimuli [35]. The parietal N20 peak is consistent and occurs contralateral to the site of stimulation [36]. Brodmann’s area 3b (the primary somatosensory cortex) responds to cutaneous inputs, but not joint movement input. Specifically, Desmedt and Osaki (1991) [27] confirmed the N20 cutaneous response in a study on passive finger movement and the absence of active joint movement. Thus, the increased amplitude of peak N20 found in experiment one is evidence of associated changes in primary somatosensory areas when learning complex tactile stimuli.

To provide context for the significance of the SEPs findings in the present study, the origin of the N24 peak is located close to the location of N20. N24 is a frontal lobe negativity that appears on the ascending slope of the N30 peak and is best revealed at high stimulus rates that selectively decrease the N30 peak [21,37]. Due to its mild variability in latency, the N24 peak has also been referred to as N23 [38] or N25 [39]. Waberski et al. (1999) [39] used source localization to identify the posterior wall of the central sulcus in area 3b of the somatosensory cortex as the site of N24 generation. In order for the pathway to be completed, the input sent to the somatosensory cortex travels through the cerebellar cortex and deep cerebellar nuclei [28]. The N24 amplitude is enhanced if the cerebellar cortex is disrupted. In contrast, the N24 amplitude is reduced or absent, but all preceding peaks are left intact if the cerebellar cortex and deep cerebellar nuclei are lesioned [40]. The characteristics of N24 are linked directly to the integrity of the cerebellum through its cortex and its deep nuclei. Experiment two yielded an attenuation of the N24 amplitude only with paresthesia. These findings provide evidence that the somatosensory integration created by a tactile response competition context is resolved in the cerebellar cortex and its deep nuclei.

The results of experiment one provide support for the phenomena known as subcortical gating [41]. Subcortical gating implies that the interference of multiple stimuli presented concurrently interacts neurologically downstream from the cortex. The increases seen in the activation at peaks N20 and N24 occurred after learning complex tactile information were equivalent between both sensory conditions. The lack of differences between the SEPs waveforms generated under different sensory conditions, in combination with the different behavioral results, provide evidence for a peripheral masking effect.

The results of experiment two provide support for the phenomena known as centripetal gating [38,41,42,43,44]. Centripetal gating is the attenuation of short latency (less than 60 ms) SEPs as a result of passive vibration or cutaneous sensory stimulation, which leads to interference between somatosensory inputs [44]. The results of experiment two provide evidence for the selective attenuation of a specific SEP component. Experimental conditions creating attenuation in single cortical peaks, but not impacting behavioral measures between conditions, reveal cortical interference in information processing. The behavioral measures revealed that no peripheral masking occurred, and the design allowed for the neurophysiological study of a response competition paradigm. The experimental conditions applied were successful in creating an interference effect that may have had ramifications for the suspected cortical generator of the N24 peak. Paresthesia presented concurrently with meaningful tactile information at separate anatomical locations suppressed peak N24. If all cortical SEPs peaks were suppressed with dual stimulation, the mechanism would be predicted to be subcortical [23]. N24 is the site considered to reflect the cerebellar role in somatosensory integration, particularly the spatial predictions or deviations of sensory input [28]. In experiment two, a significant central change occurred with the response competition design. Thus, the conflict regarding response selection was observed at the cortical level.

A possible limitation of the neurophysiological aspect of the study design was the SEPs stimulus rate of 4.98 Hz. While it enhances the accuracy of measuring cortical peak N24, it may do so by attenuation of other SEPs peaks [20,21], particularly the N30 peak, which may be why no N30 changes were found for the present experiments.

According to Maugueire (2005) [36], early SEPs waveform components (<40 ms) generated by upper limb stimulation should not be impacted by voluntary attention. Therefore, the present study’s findings address early plastic changes associated with tactile learning concurrent with competing paresthesia stimulation at spatially different locations, not changes in volitional attention. The waveform changes reflect neuroplastic changes that are evidence of response competition independent of attention.

It is not yet possible to infer if the detected neurophysiological activations result in an overall net faciliatory or inhibitory effect on behavior. Future studies building upon the knowledge generated by the presented experiments may use the SEPs peaks’ suspected cortical origins as a starting point to indicate regions to study paresthesia competing with complex tactile learning in imaging studies using methods such as fMRI and magnetoencephalography (MEG). The current experimental findings may also serve as a future model for clinician–scientists to quantify masking of information acquisition as a ramification of altered somatosensory processing of individuals with various disorders that manifest clinical paresthesia.

## 5. Conclusions

In summary, the results of the current experiments present evidence that induced paresthesia acts as a peripheral mask when presented to overlapping anatomical stimulation areas and as a central mask when the paresthesia stimulus is presented at a separate anatomical area. These findings serve to advance our understanding of the underlying neurophysiologic mechanisms of masking and response competition phenomena as they relate to complex tactile learning. Extrapolation of the information derived from the results can be a catalyst to future studies regarding the use of informative tactile stimuli in human-technology interfaces. Clinically, the results serve as a basis for future exploration of the challenges sensory impaired populations may face using today’s technology that increasingly relies on tactile cueing.
